# From Laboratory Accuracy to Industrial Deployment: A Review of Hyperspectral Imaging for Cereal Grain Quality and Safety Assessment

**DOI:** 10.3390/foods15193520

**Published:** 2026-10-01

**Authors:** Lingbo Zhou, Jichao Zhang, Guobin Zhang, Can Wang, Qiang Zhao, Mingbo Shao, Liyi Zhang

**Affiliations:** 1 Institute of Upland Food Crops, Guizhou Academy of Agricultural Sciences, Guiyang 550006, China; zhoulengbo@163.com (L.Z.);; 2 College of Agriculture, Guizhou University, Guiyang 550025, China

**Keywords:** hyperspectral imaging (HSI), cereal grain, quality assessment, non-destructive detection, deep learning, mycotoxin

## Abstract

Hyperspectral imaging (HSI) is a powerful non-destructive tool for cereal grain quality and safety assessment, enabling simultaneous prediction of multiple quality parameters through integrated spectroscopy and spatial imaging. While conventional chemometric methods such as partial least squares regression remain widely used, deep learning, particularly convolutional neural networks and ensemble methods, has demonstrated superior performance and is increasingly adopted. This review synthesizes HSI applications across six major cereal crops (maize, rice, wheat, barley, sorghum, and millet), covering nutritional composition, moisture, mycotoxins, physical traits, and variety classification. Spectral principles, modeling approaches, and cross-crop patterns are analyzed. Critical gaps are identified, including the near-absence of lipid and dietary fiber studies and scarce industrial deployment validation. Publication bias and methodological heterogeneity across primary studies, which likely inflate reported prediction accuracies, are discussed. This review provides a comprehensive reference and offers perspectives on advancing HSI toward standardized and industrially deployable solutions.

## 1. Introduction

### 1.1. Cereal Grain Quality and Traditional Assessment

Cereal grains, including maize, rice, wheat, barley, sorghum, and millet, provide approximately half of global caloric intake and serve as the foundation of both human nutrition and livestock feed [1,2]. Grain quality encompasses nutritional composition (protein, starch, amylose/amylopectin ratio, lipid, and dietary fiber), safety parameters (e.g., mycotoxin contamination, pesticide residues, heavy metals, and other chemical contaminants), physicochemical and processing-related attributes (moisture content, kernel hardness, flour yield, dough strength, and gluten content), and sensory attributes. The global grain trade, amounting to roughly 490 million tonnes per year, depends on rapid and reliable quality assessment at every point along the supply chain, from breeding programs and harvest reception to storage, processing, and export [3,4].

Conventional grain quality assessment relies primarily on wet-chemistry methods, including Kjeldahl digestion for protein, Soxhlet extraction for fat, and enzymatic assays for starch, standardized by organizations such as AACC International and ICC. While well-established, these methods are time-consuming, requiring substantial time for sampling, sample preparation, and laboratory analysis; they are also destructive and provide bulk-average measurements that mask within-sample heterogeneity [5,6]. In breeding programs, where thousands of small-volume samples must be screened annually for multiple traits, and in real-time grain storage monitoring, these limitations are particularly acute.

### 1.2. Hyperspectral Imaging as a Non-Destructive Alternative

Hyperspectral imaging (HSI) addresses these limitations by combining the chemical sensitivity of spectroscopy with the spatial resolution of digital imaging. A typical HSI system acquires a three-dimensional data cube (x, y, λ), where each pixel contains a full reflectance or transmittance spectrum across tens to hundreds of contiguous wavelength bands, enabling simultaneous chemical quantification and spatial characterization from a single acquisition [6,7,8]. The fundamental principle underlying HSI for grain quality is that specific chemical bonds (O–H, N–H, C–H, C=O, and C–O) in grain constituents absorb electromagnetic radiation at characteristic wavelengths; mycotoxins, while present at trace concentrations, induce secondary spectral changes in grain composition that can be detected through chemometric modeling [9,10]. For accurate quality prediction, the key is to develop regression and classification models that link the spectral and spatial features extracted from HSI data to quality attributes. While conventional chemometric methods such as partial least squares regression remain widely used, deep learning approaches, particularly convolutional neural networks, have demonstrated superior performance and are increasingly adopted.

### 1.3. Scope and Contribution of This Review

Several reviews have addressed specific aspects of HSI in grain science: Ma et al. [5] focused on wheat protein prediction; Femenias et al. [9] and Kabir et al. [10] reviewed mycotoxin detection; An et al. [1] surveyed AI integration with infrared spectroscopy; and Liang et al. [11] provided a broad overview of grain quality and safety. However, no prior review has synthesized HSI applications across all six major cereal crops covering the full range of quality parameters within a single framework. Across the reviewed literature, HSI consistently achieves prediction accuracy approaching that of reference wet-chemistry methods for major cereal crops; however, reported performance appears inflated by publication bias, methodological heterogeneity, and near-exclusive reliance on optimized laboratory conditions rather than industrial settings. This review synthesizes HSI applications across maize, rice, wheat, barley, sorghum, and millet, covering nutritional composition, moisture, mycotoxins, physical traits, and variety classification. The spectral principles, modeling approaches, and cross-crop patterns are analyzed, and critical research gaps are identified.

## 2. Literature Search and Scope

A literature search was conducted across Scopus, Web of Science, PubMed, and Google Scholar for English-language peer-reviewed articles published from 2021 to 2026, complemented by seminal earlier works. Multiple keyword combinations were used to capture the breadth of HSI applications in cereal grain quality assessment. Core terms included “hyperspectral imaging” combined with individual crop names (wheat, maize, rice, sorghum, barley, and millet) and quality parameters included protein content, starch content, moisture level, mycotoxins (such as aflatoxin and deoxynivalenol), lipid content, fiber content, kernel hardness, and variety classification. Supplementary searches combined “hyperspectral imaging” with method-specific terms (convolutional neural network, deep learning, partial least squares, chemometrics, support vector machine, and random forest) to ensure coverage of modeling approaches. Reference lists of relevant articles and relevant reviews were screened to identify additional records. The retrieved studies spanned all six crops and were screened for relevance before synthesis.

## 3. HSI Principles and Spectral Signatures for Grain Quality

### 3.1. HSI System and Acquisition Modes

An HSI system integrates a light source (typically tungsten-halogen for Vis-NIR or quartz-tungsten-halogen for SWIR), an imaging spectrograph, and a detector array (CCD or InGaAs) to produce a three-dimensional hypercube (x, y, λ) in which each pixel contains a full reflectance or transmittance spectrum across 100–300 contiguous wavelength bands [8]. Two acquisition modes dominate grain quality applications. Reflectance mode captures light reflected from the grain surface and is suited to color, physical defects, and surface contamination detection. Transmittance mode, in which light passes through individual kernels, penetrates the kernel interior and is preferred for internal compositional traits such as protein and moisture in single-kernel analysis [12].

### 3.2. Spectral Ranges and Key Signatures

Three principal spectral ranges are employed in cereal HSI research, each with distinct capabilities and limitations for grain quality assessment. The visible to near-infrared range (Vis-NIR, 400–1000 nm) captures electronic transitions and high-order vibrational overtones; it is sensitive to grain color, physical defects, and surface characteristics, making it suitable for variety classification and defect detection [13,14]. The near-infrared range (NIR, 900–1700 nm) captures the first and second overtones of O–H, N–H, and C–H stretching vibrations, providing the most commonly used spectral window for quantitative prediction of protein, moisture, starch, and oil content [5,15]. The short-wave infrared range (SWIR, 1000–2500 nm) accesses fundamental and first-overtone combination bands and is critical for mycotoxin detection because fungal infection alters the protein, carbohydrate, and moisture matrix of the kernel in ways that are spectrally detectable across the 1100–2500 nm region [9,10]. Full-range systems (400–2500 nm) combining Vis-NIR and SWIR coverage have been increasingly adopted for simultaneous assessment of physical and chemical quality traits [8,11]. The main spectral ranges and their representative applications in cereal grain assessment are summarized in Table 1.

**Figure 1:**
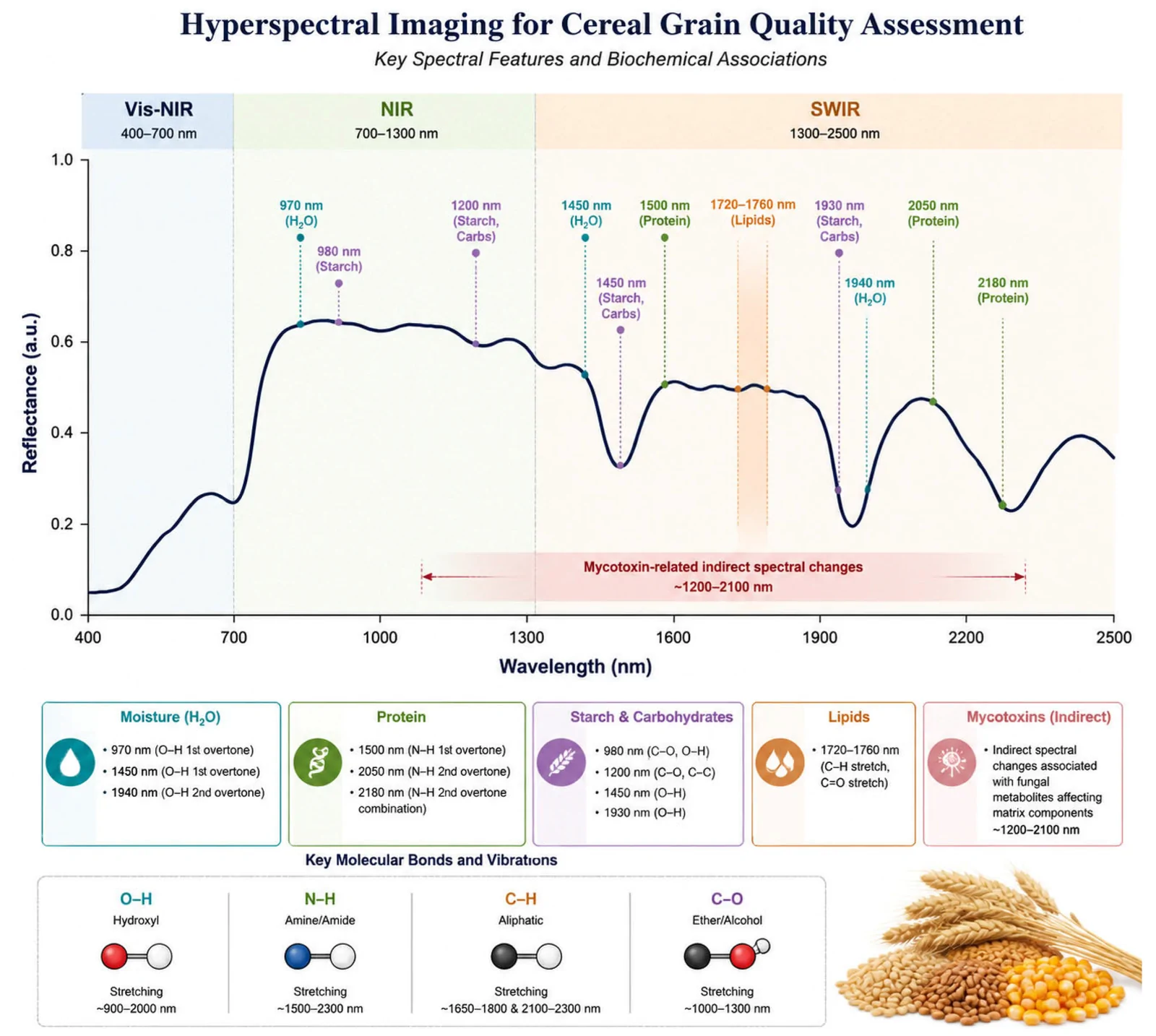
Key spectral features associated with major biochemical components in cereal grains.

The primary absorption features exploited for grain quality prediction are summarized in Figure 1. Protein exhibits characteristic N–H absorption bands near 1500, 2050, and 2180 nm; water dominates at 970, 1450, and 1940 nm; lipids display C–H stretching overtones near 1720–1760 nm; and starch and carbohydrates show broad O–H and C–O features across 980–1930 nm [8,11]. Mycotoxin detection via HSI operates through indirect mechanisms: fungal colonization alters kernel composition, and the resulting spectral changes, though subtle, can be extracted through chemometric or machine learning models [9,10,26].

## 4. HSI Applications by Quality Parameter

### 4.1. Nutritional Composition

Protein content is the most extensively studied grain quality parameter in HSI research, driven by its economic significance for baking, malting, and nutritional value. Wheat protein prediction has achieved the highest maturity: single-kernel NIR HSI was pioneered by Caporaso et al. [12], and Ma et al. [5] comprehensively reviewed HSI-based wheat protein prediction across diverse spectral ranges and modeling approaches. CNN-based methods have outperformed PLSR for wheat protein prediction [27], hybrid wavelength selection with spectral binning has further improved accuracy [28], and VIS-NIR HSI with deep learning has enabled high-throughput visualization of nutrients in wheat grains [18]. In maize, integrating spectral features with image texture features improved prediction over spectral-only models [14]. For rice, multi-spectral fusion with deep learning outperformed single-modality methods for protein prediction in brown rice [29], and rice storage proteins, including prolamin and glutelin, have been quantified using HSI with feature selection [30]. Applications in sorghum and millet are emerging, including foxtail millet protein estimation [31], essential amino acid profiling [32], carbohydrate prediction [33], and seed detection and classification in proso millet [34], but are constrained by smaller sample sizes and limited varietal diversity. Barley protein prediction has been driven by malting quality requirements, with near-infrared spectral approaches contributing to malting-quality prediction [35].

Starch and amylose content have received comparatively less HSI attention. Sorghum amylose/amylopectin prediction achieved strong predictive performance, with RPD values of 4.8–5.6 [36], and Raman HSI has emerged for maize starch detection as an alternative modality [25]. Amylose prediction performance consistently lags behind protein prediction, likely reflecting the more complex spectral expression of starch crystallinity and lower within-crop variability.

HSI studies targeting lipid and dietary fiber content remain exceedingly rare. Despite well-characterized C–H absorption features (1720–1760 nm) and demonstrated feasibility of conventional NIR spectroscopy for both parameters, HSI studies addressing lipid or fiber as the primary research focus remain exceedingly rare. This represents a significant research gap, particularly as whole-grain nutritional quality gains market importance.

### 4.2. Moisture Content

Moisture content is the most straightforward quality parameter for HSI, owing to the strong O–H absorption bands at 970, 1450, and 1940 nm. In the reviewed studies, moisture prediction routinely achieves R^2^ values above 0.95 across grain types, with RMSEP below 0.5% moisture [3]. Recent work has addressed the common challenge of limited training data through data augmentation using Wasserstein GANs [21] and HSI combined with machine learning has been applied to wheat quality monitoring during hot air drying [37]. Moisture is frequently included as a secondary output in multi-parameter models because correcting for moisture improves other predictions by accounting for spectral baseline shifts. The pressing challenge is no longer prediction accuracy but integration into real-time industrial platforms for grain drying, storage monitoring, and receipt testing.

### 4.3. Mycotoxin and Contamination Detection

Mycotoxin detection is the fastest-growing and most societally impactful HSI application in grain quality assessment. Contamination by deoxynivalenol (DON), aflatoxins, zearalenone, fumonisins, and ochratoxin A poses direct health risks and imposes substantial economic costs through rejected shipments [17,38,39].

For DON in wheat, close-range HSI discriminated contaminated from clean samples at regulatory-relevant thresholds [20]. Marín et al. [20] developed a practical framework for monitoring mycotoxin mitigation during grain processing, and Concepcion et al. [40] demonstrated the synergy of genomic prediction with HSI phenotyping for breeding DON-resistant wheat. Aflatoxin detection in maize has progressed from early proof-of-concept [16] to advanced deep learning architectures for toxin-related risk screening [41]. The demonstration that germ orientation significantly affects classification accuracy represents a methodological contribution often neglected in other studies [15]. Ergot alkaloid detection in flour streams, validated by Vermeulen et al. [42], represents one of the few HSI applications approaching regulatory-grade validation.

A critical limitation is that HSI detects compositional changes induced by fungal colonization rather than the toxin molecule itself, and direct mycotoxin quantification at regulatory (ppb) levels is generally not feasible with current HSI systems, with reported discrimination thresholds typically in the mg/kg range [9,10]. Furthermore, mycotoxin classification studies overwhelmingly report overall accuracy without confusion matrix components. Given the low prevalence of contaminated kernels, overall accuracy alone can be dangerously misleading. Future studies should report sensitivity and specificity at regulatory-relevant thresholds as minimum performance indicators, and performance should be stratified by contamination level relative to regulatory limits since early-stage contamination, precisely where screening is most needed, is where the indirect HSI signal is weakest.

### 4.4. Physical Quality Traits

HSI offers particular advantages for physical quality assessment because its spatial resolution enables pixel-level identification of defective kernels within a bulk image. Automated inspection of imperfect wheat grains, covering six defect types in addition to sound grains, has been demonstrated [43]. For rice, combined Vis/NIR/SWIR characterization [44] and deep learning regression for milling quality prediction [45] have extended HSI to processing quality assessment. Insect infestation detection has been reported for wheat and rice through spectral changes associated with internal feeding damage [46,47]. However, hardness prediction remains limited by the indirect, environment-dependent relationship between kernel mechanical properties and spectral features.

### 4.5. Variety Classification, Origin Traceability, and Other Parameters

Variety classification is the most technically mature HSI application for grains, with accuracies routinely exceeding 90% and approaching 99%. Deep learning architectures, including AlexNet for sorghum [13], dual-channel feature fusion for wheat [48], SE-enhanced 1D-CNN for maize [19], and residual Mamba architectures [49], have been successfully deployed across crops, and Raman HSI has also been applied to wheat variety identification [23]. The primary limitations are practical rather than technical: models trained on one season may degrade across seasons owing to environmental effects on grain composition. For origin traceability, multivariate curve resolution combined with Vis-NIR HSI has been applied to rice authentication [50], and spectral imaging combined with a group attention network has been used for millet origin identification [51]; millet variety identification has also been demonstrated using HSI [52].

Pre-harvest sprouting detection has been demonstrated with high accuracy using NIR HSI and deep convolutional networks [53], and Fusarium-damaged kernel detection has been reported using SWIR HSI [22]. Gluten strength and dough rheology have been explored in limited wheat studies using combined hyperspectral–RGB modeling with multi-task deep learning, reflecting the indirect nature of gluten functionality [54]. For β-glucan, a soluble dietary fiber of nutritional importance in barley and oats, no dedicated HSI study with quantitative calibration results was identified.

## 5. Methodological Approaches

### 5.1. Data Preprocessing

Raw hyperspectral data require preprocessing to remove non-chemical sources of variance. The standard pipeline includes reflectance calibration against a white reference standard; region of interest selection via automated thresholding or watershed segmentation; spectral preprocessing using standard normal variate, multiplicative scatter correction, or Savitzky–Golay derivatives to reduce scattering effects; and wavelength selection to identify the most informative bands. The choice of preprocessing strategy significantly affects model performance, yet no consensus pipeline exists; studies typically compare multiple preprocessing combinations and report the best result, introducing a risk of dataset-specific overfitting [55].

### 5.2. Chemometric and Deep Learning Methods

Partial least squares regression (PLSR) remains the most widely used method in grain HSI studies, valued for its simplicity, interpretability, and robustness with small-to-medium datasets [1]. Support vector machines address non-linearity through kernel functions and typically match or modestly outperform PLSR for classification tasks, though performance is sensitive to hyperparameter tuning. Random forest has grown in popularity for its robustness to noisy spectral data and inherent protection against overfitting through ensemble aggregation.

**Figure 2:**
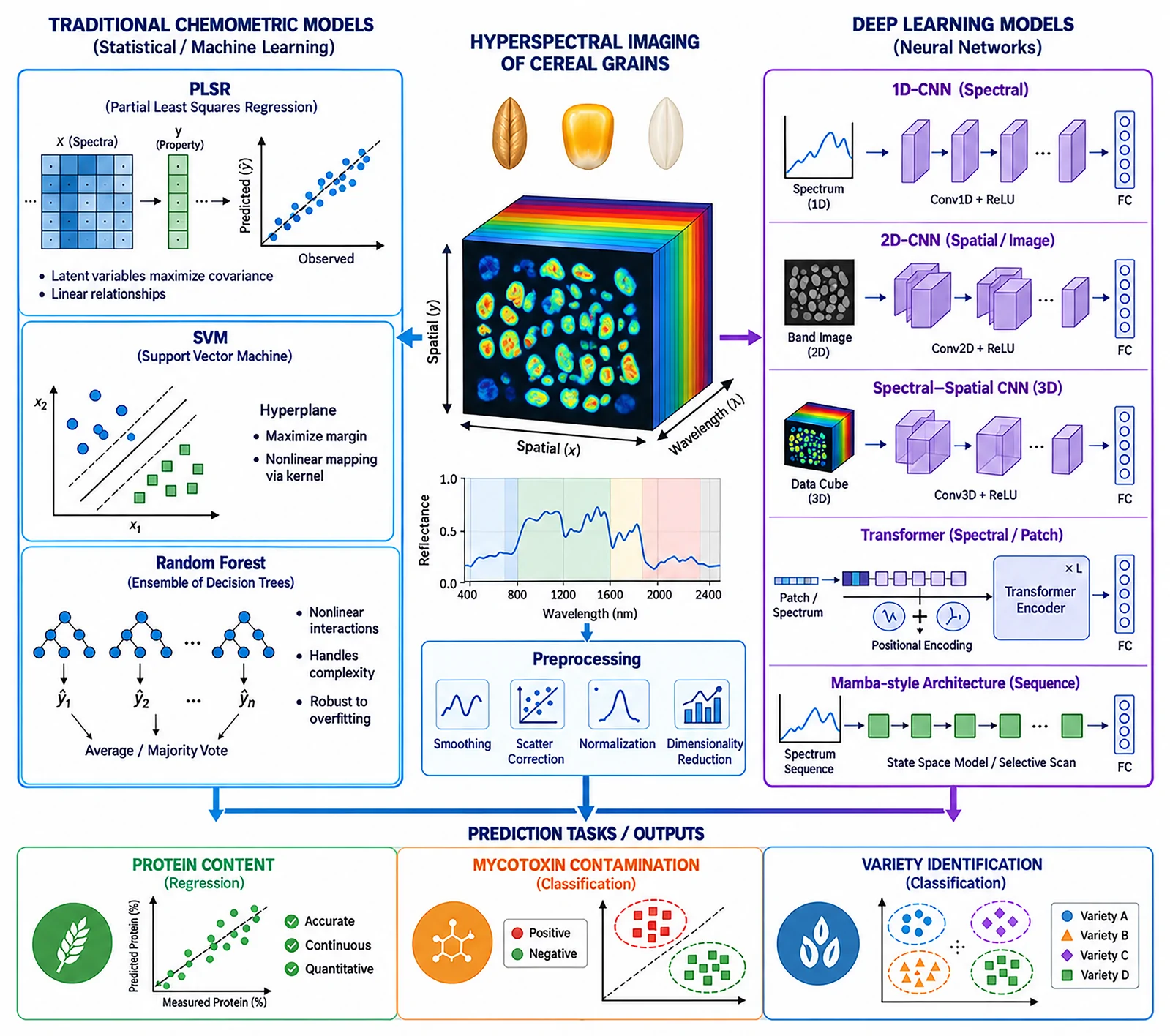
Analytical framework of traditional chemometric and deep learning models for hyperspectral imaging-based cereal grain quality assessment.

Figure 2 provides an overview of the analytical framework commonly adopted in hyperspectral imaging (HSI) studies of cereal grains. Traditional chemometric approaches, including PLSR, support vector machine (SVM), and random forest (RF), primarily rely on manually engineered spectral features after spectral preprocessing, whereas deep learning models automatically learn hierarchical spectral and spatial representations directly from hyperspectral data cubes. The workflow also highlights the typical preprocessing procedures and representative regression and classification tasks in grain quality assessment. Representative studies reviewed here have employed conventional chemometric and machine-learning approaches, including PLSR, SVM/SVR, and random forest [14,17,44,45], as well as CNN-based deep-learning architectures [15,19,27,48] and the more recent Mamba-based architecture [49]. These studies provide representative examples of the modeling approaches summarized in Figure 2.

The most significant methodological trend is the rapid adoption of deep learning, particularly convolutional neural networks (CNNs), which learn both spectral and spatial-spectral features directly from hyperspectral data. 1D-CNN has outperformed PLS-DA for fungal detection [26]; CNN regression outperformed PLSR for protein and starch prediction in brewing wheat [27]; and AlexNet and dual-channel fusion architectures have been applied to sorghum and wheat variety classification [13,14]. Ensemble methods combining multiple model types often yield the highest accuracy by leveraging complementary algorithmic strengths [56]. Transfer learning, or fine-tuning models pre-trained on one grain type for another, represents a largely unexplored opportunity with critical advantages for under-studied crops [57].

A consistent finding is that deep learning outperforms chemometrics in most published comparisons, with a typical advantage of 3–10% in R^2^ for quantitative traits. However, this advantage is not uniform: for moisture prediction, where the spectral signal is dominated by strong water absorption bands, PLSR remains competitive. The extent to which reported superiority reflects genuine advancement versus overfitting to small datasets is difficult to assess without independent external validation, which few studies performed.

### 5.3. Performance Metrics and Comparative Assessment

R^2^ was the primary performance metric in the majority of studies reviewed. While R^2^ describes variance explained, it is insensitive to systematic bias: a model with R^2^ = 0.95 and bias = 0.30% protein would consistently overestimate protein content. Only a minority of quantitative studies reported bias alongside R^2^ and RMSEP, and very few reported the ratio of performance to deviation (RPD = SDreference/RMSEP), the most interpretable metric for practical applicability [58]. Based on established guidelines, RPD values below 1.5 indicate insufficient predictive power; RPD 1.5–2.0 permits qualitative discrimination only; RPD 2.0–2.5 supports approximate quantitative prediction; RPD 2.5–3.0 indicates good quantitative prediction; and RPD above 3.0 indicates excellent quantitative prediction. For classification tasks, particularly mycotoxin detection where false negatives carry food safety consequences, performance is almost exclusively reported as overall accuracy without confusion matrix components. In an imbalanced dataset with 5% contamination, a model classifying all samples as clean achieves 95% accuracy while missing every contaminated kernel. To support practical interpretation and comparability across studies, Table 2 lists the minimum information that cereal grain HSI studies should report.

Although individual studies reported performance using different evaluation metrics and experimental protocols, several consistent trends emerge across the major quality assessment tasks. Protein and moisture prediction generally achieve the highest accuracy owing to strong spectral absorption features associated with N–H and O–H functional groups, respectively. In contrast, starch and amylose prediction remain more challenging because of overlapping carbohydrate absorption bands and interactions with other grain constituents. For classification tasks, hyperspectral imaging has demonstrated excellent capability for variety discrimination and physical defect detection, while the performance of mycotoxin detection is more variable due to the indirect nature of toxin-related spectral changes. Because absolute performance metrics are not directly comparable across studies using different datasets, spectral ranges, preprocessing strategies, and validation protocols, model comparison was focused on representative studies in which multiple algorithms were evaluated under the same experimental framework. These within-study comparisons are summarized in Table 3.

The within-study comparisons demonstrate that no single model family is uniformly superior across cereal HSI applications. Deep-learning approaches can provide substantial gains when nonlinear spectral or spectral–spatial relationships are effectively exploited, as demonstrated by CNN-based models for wheat composition and maize variety classification [27,49]. However, increased model complexity does not necessarily translate into improved predictive performance. For rice quality prediction, the optimal model varied among target traits, while PLSR retained a substantial advantage in computational efficiency [45]. Similarly, for multi-stage fungal contamination classification, LDA outperformed more complex neural-network architectures under the optimal preprocessing condition [22]. These findings indicate that model selection should consider the target property, data structure, preprocessing strategy, sample size, and computational cost rather than model complexity alone.

## 6. Cross-Crop Comparative Analysis

Wheat and maize together account for the majority of HSI grain quality publications identified in this review, reflecting their global economic significance and well-established research programs. Wheat research has concentrated on protein content, DON detection, and variety classification; maize research has broader parameter coverage with notable strengths in moisture prediction, starch content, and single-kernel analysis [59].

Rice HSI research is shaped by a distinctive quality paradigm emphasizing physical appearance (chalkiness, whiteness, grain dimensions), variety authentication, and amylose content, the primary determinant of cooking and eating quality. Aroma compounds such as 2-acetyl-1-pyrroline, responsible for the characteristic fragrance of premium varieties, represent an unexplored frontier for HSI-based prediction with significant commercial relevance.

Sorghum and millet are the most under-studied cereals relative to their global importance. Sorghum is the fifth most produced cereal globally and a staple for over 500 million people in semi-arid regions, yet HSI studies number fewer than ten. A notable domain-specific gap is tannin content, absent from the HSI literature despite profound effects on protein digestibility, nutritional bioavailability, and processing quality; conventional NIR spectroscopy has demonstrated feasibility. Millet research is even more limited; given its climate resilience and growing recognition as a nutritious alternative, rapid quality assessment tools represent an urgent need.

Barley HSI research is concentrated almost exclusively on malting quality parameters driven by the brewing industry. NIR-based approaches, including integration of spectral data with genomic prediction, have been applied to barley variety identification and malting quality [35]. Whole-grain barley for food applications has received negligible HSI attention.

Across all crops, research intensity correlates strongly with economic value in international grain trade rather than with global food security importance. The cross-crop evidence is summarized in Table 4.

## 7. Challenges and Research Gaps

### 7.1. Publication Bias and Methodological Heterogeneity

Studies reporting successful predictions dominated the literature. No study reporting failure to predict a quality parameter with useful accuracy was identified. Consequently, the performance metrics summarized in this review and in the primary literature likely represent upper-bound estimates rather than expected field performance. Compounding this issue is extreme methodological heterogeneity in acquisition parameters, preprocessing pipelines, modeling algorithms, and validation protocols, which impedes both cross-study comparison and the development of consensus best practices.

### 7.2. Knowledge Gaps: Unexplored Parameters and Under-Studied Crops

The most conspicuous knowledge gap is the absence of HSI studies targeting lipid and dietary fiber content in cereal grains, despite well-established NIR absorption features and demonstrated non-imaging NIR feasibility. Sorghum tannin content, with significant nutritional and processing implications, has received no HSI attention despite clear spectroscopic feasibility. Barley, sorghum, millet, oats, and rye are substantially under-studied relative to their global production volumes. For millet, a climate-resilient crop increasingly promoted for sustainable agriculture, published HSI quality studies remain very limited. Targeted research programs applying established HSI methodologies to these crops would yield disproportionate benefits.

### 7.3. The Laboratory-to-Industry Translation Gap

**Figure 3:**
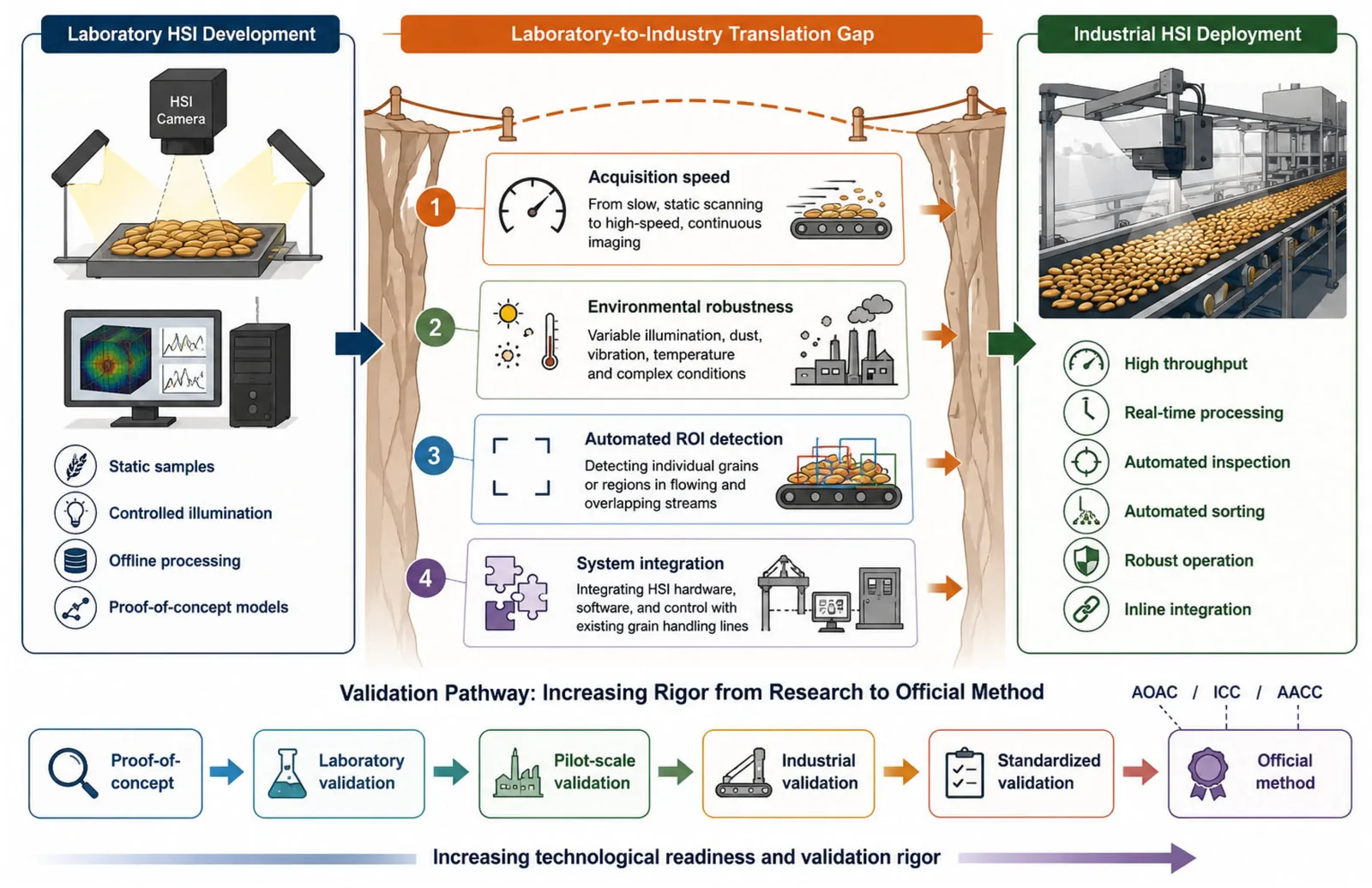
From laboratory HSI model development to industrial deployment: key barriers and validation requirements.

The overwhelming majority of grain HSI studies are conducted under laboratory conditions with static samples, controlled lighting, and offline data processing. Translating these into industrial-grade systems requires solving engineering challenges in acquisition speed, robustness to variable ambient conditions, automated ROI detection for flowing grain streams, and integration with existing grain handling infrastructure [3]. Very few studies report industrial deployment validation, and no HSI-based method has achieved AOAC, ICC, or AACC official method status. The ergot detection validation by Vermeulen et al. [42] represents the closest example of a method approaching this level of rigor. Figure 3 illustrates the principal barriers between laboratory HSI model development and industrial deployment, together with the validation levels required to progress along this pathway.

## 8. Future Directions

Several technological developments could reshape grain HSI in the coming years. Transformer-based architectures and state-space models such as Mamba enable more powerful spectral feature learning with improved scalability; their systematic evaluation for grain quality prediction beyond the proof-of-concept demonstrated by Qi et al. [49] is warranted. Self-supervised pre-training approaches represent a high-priority research direction, as learning general spectral representations from large unlabeled datasets before fine-tuning on small labeled sets could reduce the labeled data requirements for minor crops by an order of magnitude. Snapshot hyperspectral cameras, which capture the full spatial-spectral hypercube in a single acquisition, offer faster acquisition at lower cost, potentially enabling industrial-speed grain quality assessment. Multi-sensor fusion platforms combining HSI with X-ray imaging, Raman spectroscopy, or multispectral imaging may surpass any single modality for comprehensive quality assessment [2,24].

For practical industrial and breeding adoption, deployment strategies should prioritize standardized calibration protocols enabling model transfer between instruments, edge computing solutions for on-camera real-time inference, and integration with genomic prediction pipelines where HSI-derived phenotypes serve as intermediate traits for genomic selection [60]. Multi-laboratory validation studies are needed to provide the evidence base for regulatory recognition. Open-access spectral databases spanning diverse genotypes, environments, and growing seasons would accelerate community progress and reduce duplicated calibration efforts.

Climate change is altering grain quality in ways that increase the urgency of rapid multi-parameter assessment. Elevated CO_2_ reduces grain protein and micronutrient concentrations in wheat and rice [61]; heat stress during grain filling reduces dough strength and increases rice chalkiness; and drought stress increases aflatoxin contamination risk in maize. HSI-based quality phenotyping connected to climate adaptation breeding programs could support selection of varieties maintaining grain quality under elevated CO_2_, heat, and drought, an application with significant food security implications.

## 9. Conclusions

Hyperspectral imaging has demonstrated significant promise for non-destructive cereal grain quality and safety assessment. This review synthesized HSI applications across six major cereal crops, covering nutritional composition, moisture, mycotoxins, physical traits, and variety classification. The evidence confirms that HSI achieves useful prediction accuracy for protein content, moisture, and variety classification in wheat, maize, and rice, with deep learning methods consistently outperforming traditional chemometrics. However, applications in sorghum, millet, and barley remain sparse, and critical gaps persist in lipid and dietary fiber prediction, cross-crop model transferability, and industrial deployment validation. The limited reporting of bias and RPD alongside R^2^, and of sensitivity and specificity in mycotoxin classification, constrains the practical interpretability of published performance metrics. Although laboratory feasibility is well established, real-world deployment remains limited; very few studies have validated HSI under industrial conditions. Future work should focus on developing standardized acquisition and reporting protocols, conducting multi-laboratory validation studies, and establishing open spectral databases to support calibration transfer across instruments and crop types. With continued progress in deep learning architectures, transfer learning, and hardware integration, HSI is expected to play an increasingly important role in advancing grain quality and safety evaluation.

## Figures and Tables

**Table 1:** Spectral ranges and representative applications of HSI in cereal grain assessment.

Spectral Range	Main Information	Typical Cereal Applications	Key Limitations	Representative Studies
Vis-NIR 400–1000 nm	Color, pigments, surface features, high-order overtones	Variety classification, defects, surface mold	Limited penetration and weaker chemical specificity	[14,15,16,17,18,19]
NIR 900–1700 nm	O–H, N–H, C–H overtones	Moisture, protein, starch, kernel quality	Overlapping bands, moisture interference	[20,21]
SWIR 1000–2500 nm	Stronger chemical absorption features	Protein, water, lipid, fungal damage, mycotoxin risk	Higher cost, slower acquisition	[12,21,22]
Raman HSI	Molecular vibration information	Starch, variety, composition	Weak signal, longer acquisition, fluorescence interference	[23,24,25]

**Table 2:** Minimum reporting checklist for cereal grain HSI studies.

Category	Minimum Information to Report	Representative Studies
Samples	Crop, variety, genotype, location, year, storage condition, sample size	[12,14,19,21,30]
Reference method	Laboratory method, measurement uncertainty, regulatory threshold if relevant	[12,17,20]
HSI system	Spectral range, resolution, imaging mode, illumination, detector, calibration	[12,14,15,17,21,22]
Data processing	ROI selection, preprocessing, feature selection, segmentation	[14,21,22,30]
Model	Algorithm, hyperparameters, baseline comparison, software	[15,19,22,27,30,43,45,48,49]
Validation	Data split unit, external test set, batch/year/location/instrument independence	[12,19,40,49]
Regression metrics	R^2^, RMSEC, RMSECV, RMSEP, MAE, bias, RPD/RPIQ	[12,17,21,27,30,36]
Classification metrics	Accuracy, sensitivity, specificity, precision, recall, F1-score, AUC, confusion matrix	[15,19,22,43,48,49]
Robustness	Repeated runs, confidence intervals, failure cases	[15,19,40,49]
Deployment	Acquisition speed, throughput, real-time feasibility, industrial environment	[20,42]

**Table 3:** Within-study comparative performance of representative modeling approaches for HSI-based cereal grain quality and safety assessment.

Ref.	Crop and Target	Models Compared	Key Within-Study Performance	Comparative Interpretation
[15]	Maize; fungal contamination classification	PLS-DA, ANN, 1D-CNN	Under train–test orientation mismatch, average error rates were 5.71%, 4.94%, and 3.15% for PLS-DA, ANN, and 1D-CNN, respectively; the germ-up 1D-CNN achieved an error rate of 1.31%.	1D-CNN showed the strongest overall discrimination and robustness, but kernel orientation substantially affected classification performance.
[27]	Wheat; protein and starch prediction	PLSR, XGBoost, CNNR	At full wavelength, CNNR provided the best prediction: protein, R^2^ = 0.9942, RMSE = 0.1041, RPD = 13.1306; starch, R^2^ = 0.9329, RMSE = 0.8633, RPD = 3.8605.	CNNR outperformed the conventional models under the full-spectrum setting, indicating an advantage in learning nonlinear spectral features without explicit wavelength selection.
[30]	Rice; prolamin and glutelin prediction	PLSR, SVR, BPNN, CNN	Full-spectrum test r/RMSEP for prolamin: PLSR 0.722/0.057, SVR 0.714/0.059, CNN 0.779/0.103, BPNN 0.831/0.051; for glutelin: 0.794/0.667, 0.828/0.598, 0.813/1.410, and 0.902/0.526, respectively.	BPNN provided the best overall prediction for both protein fractions; CNN did not consistently outperform conventional regression models.
[45]	Rice; BRR, MRR, and HRR prediction	PLSR, SVR, CNN, BPNN	In multi-task/multi-output prediction, the best test results were SVR for BRR (r_p_ = 0.865, RMSEP = 0.281) and BPNN for MRR (r_p_ = 0.819, RMSEP = 0.421) and HRR (r_p_ = 0.870, RMSEP = 0.766). Training time ranged from 3.143 s for PLSR to 1953.404 s for BPNN.	No single architecture dominated all targets. BPNN showed stronger overall prediction, whereas PLSR offered a substantial computational-efficiency advantage.
[49]	Maize/variety	SVM, ELM, BP, LSTM, 1D-CNN, Res1DCNN, Mamba1DCNN, RM1DNet	Average accuracy: 70.57, 59.12, 57.21, 85.25, 94.14, 94.39, 94.57, and 94.85%, respectively	Deep spectral models substantially outperformed conventional ML; RM1DNet performed best
[53]	Wheat; pre-harvest sprouting classification	1D-CNN, 2D-CNN, 3D-CNN, mixed CNN	Test accuracies were 96.81%, 96.02%, 98.40%, and 98.12%, respectively.	3D-CNN achieved the highest accuracy, whereas the mixed CNN achieved nearly comparable performance with fewer trainable parameters, illustrating an accuracy–complexity trade-off.
[22]	Maize/fungal contamination	LDA, PCA-LDA, SVM, MLP, CNN/LSTM/Transformer hybrid	Binary: SNV-MLP 100%; six-class: SD-LDA 92.56%	More complex deep architectures did not universally outperform simpler models
[54]	Wheat; protein and dough rheological traits	PLSR, XGBoost, MTL-AM	Using fused wavelet features and color indices, R^2^ values for PLSR/XGBoost/MTL-AM were 0.875/0.963/0.972 (GPC), 0.881/0.948/0.969 (WGC), 0.848/0.952/0.968 (WA), 0.817/0.932/0.970 (WD), and 0.765/0.917/0.901 (FQN).	MTL-AM performed best for four of five traits, whereas XGBoost was superior for FQN, confirming that model superiority remains target-dependent.

**Table 4:** Cross-crop evidence matrix of HSI applications for cereal grain quality and safety assessment.

Crop	Main Targets	Evidence Maturity	Main Gaps	Industrial Readiness
Wheat	Protein, DON, Fusarium, defects, variety	High	External validation, industrial sorting, multi-year data	Moderate
Maize	Moisture, starch, protein, fungal contamination, aflatoxin risk	High	Kernel orientation, toxin confirmation, online validation	Moderate
Rice	Chalkiness, milling quality, amylose, protein fractions, authenticity	Moderate	Aroma, external validation, processing-line testing	Low to moderate
Sorghum	Variety, starch, amylose, protein	Low to moderate	Tannin, food safety, large genotype panels	Low
Millet	Variety, protein, carbohydrate, seed classification	Low	Fiber, lipid, minerals, storage safety, multi-species datasets	Low
Barley	Protein, malting traits, variety	Low to moderate for HSI	β-glucan, food-use traits, HSI-specific validation	Low

## Data Availability

No new data were created or analyzed in this study. Data sharing is not applicable to this article.
